# Multi-Scenario Simulation to Predict Ecological Risk Posed by Urban Sprawl with Spontaneous Growth: A Case Study of Quanzhou

**DOI:** 10.3390/ijerph192215358

**Published:** 2022-11-21

**Authors:** Jiangfu Liao, Lina Tang, Guofan Shao

**Affiliations:** 1 Computer Engineering College, Jimei University, Xiamen 361021, China; 2 Key Lab of Urban Environment and Health, Institute of Urban Environment, Chinese Academy of Sciences, Xiamen 361021, China; 3 Department of Forestry and Natural Resources, Purdue University, West Lafayette, IN 47907, USA

**Keywords:** cellular automata, urban sprawl, spontaneous growth, scenario simulation, ecological risk

## Abstract

The rapid expansion of different types of urban land continues to erode natural and semi-natural ecological space and causes irreversible ecological damage to rapidly industrialized and urbanized areas. This work considers Quanzhou, a typical industrial and trade city in southeastern China as the research area and uses a Markov chain integrated into the patch-generating land use simulation (PLUS) model to simulate the urban expansion of Quanzhou from 2005 to 2018. The PLUS model uses the random forest algorithm to determine the contribution of driving factors and simulate the organic and spontaneous growth process based on the seed generation mechanism of multi-class random patches. Next, leveraging the importance of ecosystem services and ecological sensitivity as indicators of evaluation endpoints, we explore the temporal and spatial evolution of ecological risks from 2018 to 2031 under the scenarios of business as usual (BAU), industrial priority, and urban transformation scenarios. The evaluation endpoints cover water conservation service, soil conservation service, biodiversity maintenance service, soil erosion sensitivity, riverside sensitivity, and soil fertility. The ecological risk studied in this work involves the way in which different types of construction land expansion can possibly affect the ecosystem. The ecological risk index is divided into five levels. The results show that during the calibration simulation period from 2005 to 2018 the overall accuracy and Kappa coefficient reached 91.77% and 0.878, respectively. When the percent-of-seeds (PoS) parameter of random patch seeds equals 0.0001, the figure of merit of the simulated urban construction land improves by 3.9% compared with the logistic-based cellular automata model (Logistic-CA) considering organic growth. When PoS = 0.02, the figure of merit of the simulated industrial and mining land is 6.5% higher than that of the Logistic-CA model. The spatial reconstruction of multiple types of construction land under different urban development goals shows significant spatial differentiation on the district and county scale. In the industrial-priority scenario, the area of industrial and mining land is increased by 20% compared with the BAU scenario, but the high-level risk area is 42.5% larger than in the BAU scenario. Comparing the spatial distribution of risks under the BAU scenario, the urban transition scenario is mainly manifested as the expansion of medium-level risk areas around Quanzhou Bay and the southern region. In the future, the study area should appropriately reduce the agglomeration scale of urban development and increase the policy efforts to guide the development of industrial land to the southeast.

## 1. Introduction

Ecological risk research focuses on analyzing the probability that threats outside the ecosystem will lead to irreversible damage to the structure and function of the ecosystem [1,2,3]. At the lower level, ecological risk assessments have focused more on the adverse effects of a single physical or chemical stress on an individual organism or a small group of representative species [4,5]. At higher levels such as populations or communities, more effective assessment of ecological risk in the context of multiple stressors and multiple ecological receptors continues to attract the attention of assessors [6,7,8]. Analyzing ecological risk based on ecosystem services can better describe the way in which external drivers stemming from human well-being can negatively impact internal ecosystem mechanisms [9,10,11]. In rapidly urbanizing areas, the loss of ecological space leads to the loss of ecological services and the degradation of ecological functions [12]. Regional ecological risk assessment involves evaluating the likelihood of adverse effects and the potential harm when ecological receptors are exposed to natural disasters or man-made disturbances in the context of specific temporal and spatial backgrounds [13,14,15,16]. Given the co-occurrence of multiple risk sources and multiple ecological receptors in the environment on a specific spatial scale, ecological risk analysis on the regional scale faces significant challenges, including complex interactions, variations in the importance and sensitivity of ecological receptors, data acquisition, uncertainty, and the involvement of numerous stakeholders [6,17].

To investigate ecological risks on the regional scale, studies have assessed the sensitivity of specific ecosystems near urban cores to pollution (such as heavy metals) produced by human activity [13]. Following the principles of efficiency of ecological modeling, several methods such as the relative risk model have been developed and used to assess ecological risks in urban areas [18,19,20,21]. High-level ecological models on the regional scale prioritize accuracy [6]. Simulating fine-scale changes in urban land use is an important way to explore the ecological risks manifested by the loss of the ecosystem services provided by an ecological space or by an increased sensitivity of the ecological environment [22,23]. However, simulations of urban expansion that consider “urban–non-urban” or land use changes involving transitions between multiple land types generally combine impervious surfaces into a single type of urban land [24,25]. Research remains sparse on the simulation of the spatiotemporal evolution of various types of construction land such as industrial land, mining land, urban construction land, and rural settlements. The observation of patch-level modification of land use reveals two types of spatial expansion, organic growth and spontaneous growth [26]. Such growth is common for all land types, especially land types involving the spatiotemporal expansion of construction land [27,28,29]. Since the spontaneous growth of different types of construction land patches may differ significantly, the simulation of urban land use scenarios involving multiple types of construction land has fundamental temporal and spatial importance for regional ecological risk assessment.

Multi-scenario simulation is an effective way to explore the way in which urbanization and industrialization affect natural ecological spaces, which helps to develop policy responses [30,31]. Compared with conventional logical reasoning and mathematical derivations, cellular automata (CA) simulations can clarify the spatiotemporal dynamics of land use and land cover change (LUCC). The dynamic simulation of urban development based on CA continues to attract research, and CA-based urban models (e.g., SLEUTH, CLUE-S, and FLUS) are widely used in fields such as ecology and environmental science [32,33,34]. Markov chains, system dynamics, and multi-objective constrained optimization are the main approaches for predicting land demand in urban CA models [35,36,37]. Leveraging accurate statistics of the transformation information between historical land uses, the Markov chain model uses the estimated transition probability matrix to predict future land demand. Since the scale of development of the dominant land use types is prone to sudden changes, the key parameters and the structure of Markov models must improve to understand the evolution of demand under multiple scenarios.

To simulate the spontaneous growth process at the patch level, this study predicts land use scenarios based on the patch-generating land use simulation (PLUS) model developed by [36]. The PLUS model uses the land use expansion analysis strategy (LEAS) based on the random forest algorithm to determine the probability that certain land uses develop. This method avoids the exponential growth that may result from traditional transformation analysis strategy in multi-type land use simulations while at the same time enhancing the ability of pattern-analysis strategy to determine the mechanisms that drive land use change. The CA model based on multi-type random patch seeds (CARS) accurately simulates the spontaneous growth dynamics of land use, thereby providing fine-resolution spatial distribution information of construction land for regional ecological risk assessment. Studies have shown that the PLUS model more accurately predicts land use than the traditional CA model [38]. Recently, the PLUS model was applied by the authors of [39] to study the level of risk posed to landscape functions and ecosystems led by different modes of urban expansion under shared socioeconomic pathways. Based on an ecological risk prediction analysis of land use change under multiple scenarios, the authors of [22] claim that in areas with dense population and concentrated construction land, the construction of ecological corridors is an important option for alleviating ecological pressure. However, few reports consider the regional ecological risk assessment while accounting for the complex spatiotemporal evolution of multiple types of urban construction land.

Quanzhou is an important starting point of the “Maritime Silk Road” in ancient China and one of the first 24 famous historical and cultural cities announced by the State Council of China. At present, Quanzhou is actively integrating into China’s One Belt One Road development strategy and continues to lead in the construction of the economic zone on the west coast of the Taiwan Strait. In 2021, Quanzhou’s GDP reached 1.13 trillion yuan, ranking it first among major cities in the Golden Triangle of Southern Fujian [40]. At present, numerous industrial clusters have formed around Quanzhou with an output value of more than 100 billion yuan, including machinery and equipment, petrochemicals, textiles and garments, paper printing, building materials, and home furnishing. The registered population of Quanzhou in 2020 was 7.6614 million, of which the agricultural population accounts for 46.70% [41]. Various types of construction land, such as urban construction land, industrial and mining land, and rural settlements, are densely distributed in the middle and lower reaches of the Jinjiang River Basin. However, Quanzhou is in the hilly red soil area of southern China, and its fragile ecological environment translates into extremely limited space for the development of construction land. The dense spatial and temporal connections among important ecological spaces, limited land resources, and socio-economic development dynamics make Quanzhou a suitable case study for regional ecological risk.

Therefore, to predict the temporal and spatial evolution of ecological risks in Quanzhou, the objectives of this work are as follows: (1) to establish the linking mechanism of “urban expansion ecological space occupation–ecosystem service damage” in regional ecological risk assessment; (2) to accurately predict the hotspot areas of land use ecological risks by using organic growth and spontaneous growth simulations of different types of construction land. We use a Markov chain integrated into the PLUS model to simulate the land use change in Quanzhou over the period 2005–2031. Next, we establish a risk assessment framework with construction land expansion as the risk source and ecosystem services and ecological sensitivity as the evaluation endpoints. Finally, we evaluate the future ecological risk in the study area on global and local scales and for different urban development scenarios.

## 2. Study Area and Experimental Data

### 2.1. Study Area

**Figure 1:**
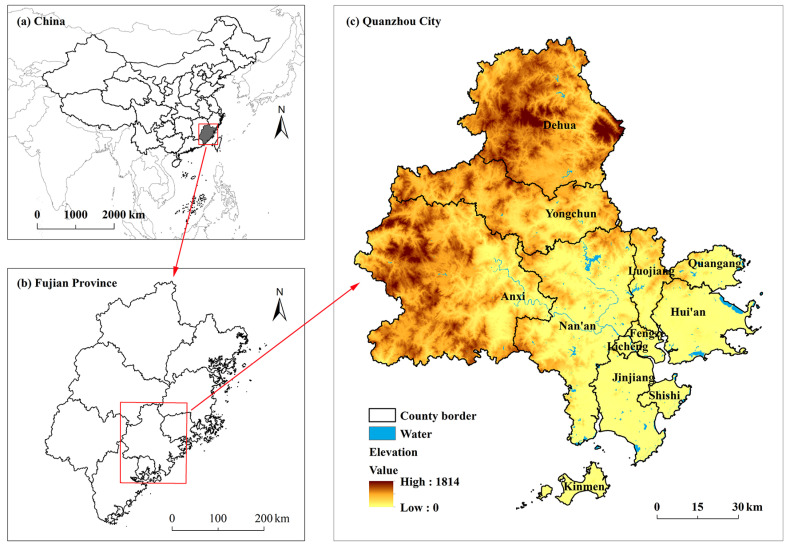
Location of the study area.

Quanzhou is located on the southeast coast of China (117°25′–119°05′ E, 24°30′–25°56′ N) and is one of the central cities on the west coast of the Taiwan Strait. It is also a modern industrial, trade, and port city. Counties and urban areas in Quanzhou City, including Quangang, Licheng, Fengze, Luojiang, Jinjiang, Shishi, Nan’an, Dehua, Anxi, Hui’an, and Yongchun, were thus selected as the study area (Figure 1). The study area contains scattered hills, valleys, and basins, with the elevation decreasing from the northwest to the southeast. Quanzhou has an area of 11,015 km^2^, with mountains and hills accounting for about four-fifths of the total area. Quanzhou has a subtropical marine monsoon climate, with an average annual temperature of about 20.3 °C and an average annual rainfall of about 1700 mm.

### 2.2. Experimental Data and Data Sources

The data in this study cover land use, driving factors, socioeconomic statistics, ecological services, and ecological sensitivity. Land use and land cover data were obtained from the Institute of Remote Sensing and Digital Earth, Chinese Academy of Sciences (http://www.aircas.cas.cn (accessed on 1 October 2022)) and include forest land, farmland, grassland, water bodies, beaches, urban construction land, industrial and mining land, and rural settlements. The driving factors are elevation, slope, distance to urban center, distance to town center, distance to highway, distance to railway, distance to main roads, distance to coastline, and distance to train station. The public budget, per capita disposable income, fixed asset investment, and other social and economic statistics were obtained from the Socio-economic Statistical Yearbook of Quanzhou City. Housing prices are based on data released by China House Price Market Network (https://m.creprice.cn (accessed on 1 October 2022)) in January 2022. The industrial zone planning comes from the Territory Development Plan of Quanzhou City (2021–2035). Geographical information system (GIS) datasets related to the evaluation of ecosystem services and ecological sensitivity are mainly composed of air temperature, precipitation, net primary productivity, and soil texture. The temperature and precipitation are China’s ground cumulative annual values (1981–2010), and the data come from the China Meteorological Data Network. Net primary productivity is derived from NASA’s MOD17A3 dataset (2005–2018). The soil texture is derived from the Chinese soil dataset of the “Big Data Center of Sciences in Cold and Arid Regions” (http://www.casnw.net (accessed on 1 October 2022)). To simulate land use change, the spatial resolution of the relevant GIS data is unified to 30 m × 30 m. The spatial grid thus formed contains 5022 columns and 5277 rows and defines the data format for the input, operation, and output of the PLUS model.

## 3. Methods

To explore the temporal evolution and spatial characteristics of regional ecological risks under the complex spatial reconstruction of multiple types of construction land, this work adopts the following technical process. First, based on the land use classification data from 2005 to 2018, the revised Markov transition probability is used to estimate the urban land demand under different development goals. Second, the random forest algorithm is used to determine the way in which the various driving factors contribute to land use change, and the PLUS model is used to predict the spatial structure of urban land use under different development scenarios. Finally, based on the proposed ecological risk assessment framework, regional ecological risks due to loss of ecosystem services and increased ecological sensitivity are predicted.

### 3.1. Revised Markov Transition Probabilities to Predict Land Demand

A Markov chain is a special stochastic motion process that describes the “no aftereffect” probability distribution upon moving from one state to another [42]. The key to predicting urban land demand using a Markov model is to construct a transition probability matrix for mutual transformation between different land uses [43]. The annual transfer rate of a certain land use type can be calculated by using observational land use data from two points in time, which gives the transition probability matrix for this period. According to the homogeneous Markov chain and Bayesian conditional probability formula, the following Markov model for predicting urban land demand can be established [44]:

(1) $$P_{\left(n\right)}=P_{\left(n-1\right)}P_{ij},$$

where $P_{\left(n\right)}$ is the state probability vector of the object being studied in the system at any time and $P_{\left(n-1\right)}$ is the preliminary state probability vector of the object under study. To increase the built-up land area by using a socioeconomic model or according to regional macro-scale land use planning, the transition probability matrices used to predict land conversion demand were further revised.

Let $A_{a}$ and $A_{m}$ be the new area of specific construction land predicted by the planning scenario and the Markov model, respectively. These two parameters often differ significantly. Although the urban expansion area that dominates urban LUCC needs to be revised, the Markov model still reflects land use and land development characteristics in a region. The probability of transition from different land use types to urban built-up land can be modified by tuning the ratio $\mu_{j}$ of $A_{a}$ to $A_{m}$. A ratio $\mu_{j}$ < 1 indicates that the conversion of other land use types to built-up land has been revised downwards, and vice versa. Additionally, to ensure that the elements of the transition probability matrix satisfy constraint (2), the probability of converting land use from land use type i to another land use types is corrected by using $\mu_{i}$, and the Markov transition probability matrix modified by the planning development goal takes the form

(2) $$P^{\prime }=\left(P_{ij}{}^{\prime }\right)=\left|\begin{array}{cccccc} \mu_{1}P_{11} & \mu_{1}P_{12} & \ldots \; & \mu_{j}P_{1j} & \ldots & \mu_{1}P_{1M}\\ \mu_{2}P_{21} & \mu_{2}P_{21} & \ldots \; & \mu_{j}P_{2j} & \ldots & \mu_{2}P_{2M}\\ \ldots & \; & & \; & & \\ \mu_{i}P_{i1} & \mu_{i}P_{i2} & \ldots \; & \mu_{j}P_{ij} & \ldots & \mu_{i}P_{iM}\\ \ldots & \; & & \; & & \\ P_{j1} & P_{j2} & \ldots \; & P_{jj} & \ldots & P_{jM}\\ \ldots & \; & & \; & & \\ \mu_{M}P_{M1} & \mu_{M}P_{M2} & \ldots \; & \mu_{j}P_{Mj} & \ldots & \mu_{M}P_{MM} \end{array}\right|,$$

where the subscript j indicates built-up land and $\mu_{j}$ and $\mu_{i}$ are the aforementioned transition probability correction coefficients. Here, the renewal of built-up land and the corresponding transition probabilities ($P_{j1},P_{j2},\ldots ,P_{jM}$) are not modified. The correction coefficient $\mu_{i}$ can be calculated as follows:

(3) $$\mu_{i}=\frac{1-\mu_{j}P_{ij}}{\sum_{k=1}^{M}P_{ik}-P_{ij}};\;\;\;\;i=1,2,\ldots ,M;\;i\neq j,$$

where k is the subscript of land use in the transition probability matrix, $P_{ik}$ is the probability of converting land use type i into land use type k, i indicates the land use type other than built-up land, and *j* indicates the specific category of built-up land.

### 3.2. LUCC Simulation with CA Model Based on Multi-Type Random Patch Seeds

The PLUS model simulates organic and spontaneous growth due to land use change primarily by using a generation strategy of multi-type random patch seeds. The simulation of land use change by this model is implemented by two components: the LEAS and the CA models based on CARS. The key objective of the LEAS is to accurately capture the way in which the driving forces contribute to the various types of land use expansion during the simulation period. LEAS first extracts the way various types of land use change between the two input land use status quos, and samples from the newly developed pixels of various types of land use. This module converts the transition rule mining of each type of land use into a binary classification problem [36] and uses the random forest algorithm to predict the probability of development of any given land use. Combined with random seed generation and the decreasing threshold mechanism, CARS establishes an adaptive competition mechanism to simulate the spontaneous growth process of land use change. CARS establishes the following method to calculate the overall probability for coupling organic growth and spontaneous growth according to whether the value of the neighborhood function of the central cell is zero:

(4) $$OP_{i,k}^{d=1,t}=\{\begin{array}{cc} P_{i,k}^{d=1}\times \left(r\times \mu_{k}\right)\times I_{k}^{t} & {\;\mathrm{if}\;\Omega }_{i,k}^{t}=0\;\mathrm{and}\;r<P_{i,k}^{d=1}{}_{}\\ P_{i,k}^{d=1}\times \Omega_{i,k}^{t}\times I_{k}^{t} & otherwise \end{array},$$

where $OP_{i,k}^{d=1,t}$ is the overall probability of land type k, $P_{i,k}^{d=1}$ is the probability of development of land type k in pixel i, $\Omega_{i,k}^{t}$ is the cover fraction of land use type k in the 3 × 3 Moore neighborhood of pixel i, r is a random number between zero and one, $\mu_{k}$ is the threshold for generating new “enclave patches” for land type k, and $I_{k}^{t}$ is an adaptive driving factor that characterizes the impact of future demand on land type k, which is calculated as follows:

(5) $$I_{k}^{t}=\{\begin{array}{c} I_{k}^{t-1}\;\;\;\;\;\;\;\;\;\;\;\;\;\;\;\;\;\;\;\;if\;\left|G_{k}^{t-1}\left|\leq \right|G_{k}^{t-2}\right|\\ I_{k}^{t-1}\times \frac{G_{k}^{t-2}}{G_{k}^{t-1}}\;\;\;\;\;\;\;\;if\;G_{k}^{t-1}<G_{k}^{t-2}<0\\ I_{k}^{t-1}\times \frac{G_{k}^{t-1}}{G_{k}^{t-2}}\;\;\;\;\;\;\;\;\;\;if\;G_{k}^{t-1}>G_{k}^{t-2}>0 \end{array}.\;\;\;\;\;$$

In Equation (5), $G_{k}^{t-1}$ and $G_{k}^{t-2}$ are the differences between the cumulative development amount and future demand of category k at iterations t−1 and t−2, respectively. Setting the driving coefficient establishes multi-directional feedback of competitive development among various land types and promotes the orderly development of various land uses. At the same time, CARS restricts spontaneous and organic growth of multiple land uses through a decreasing threshold mechanism of a competitive process. The overall probability of all land uses $OP_{i,k}^{d=1,t}$ as input into a roulette mechanism to select candidate land use type c for pixel i. Next, whether the pixel transitions to land use type c is assessed as follows by a decreasing threshold τ:

(6) $$\mathrm{If}\;\sum_{k=1}^{N}\left|G_{c}^{t-1}\right|-\sum_{k=1}^{N}\left|G_{c}^{t}\right|<Step,\;\;\mathrm{then}\;l=l+1,$$

(7) $$\{\begin{array}{c} Change\;\;\;\;\;\;\;\;\;\;\;\;\;\;\;\;if\;P_{i,c}^{d=1}>\tau \;\;\mathrm{and}\;TM_{k,c}=1_{}\\ No\;change\;\;\;\;\;\;\;\;\;\;if\;P_{i,c}^{d=1}\leq \tau \;\;\mathrm{and}\;TM_{k,c}=0_{}\; \end{array}\;\;\;\;\;\tau =\delta^{l}\times r,$$

where Step is the step length of CARS approaching land demand, l is the number of decay steps for automatic adjustment, $TM_{k,c}$ is the cost matrix that allows the conversion of land use type k to c [45], δ is a customizable attenuation factor that varies from zero to one, and r is a normally distributed random value between zero and two with a mean of one. In this research, the patch generation threshold *δ* is set to 0.1, and the expansion coefficient $\mu_{k}$ that determines the probability of random patch seeds is set to 0.9. Recently, an improved version of CARS added a parameter “percent-of-seeds” (PoS) that controls the number of random seeds and ranges from zero to one.

We evaluate the accuracy of PLUS simulations based on the overall accuracy, Kappa coefficient, and figure of merit (FoM). The overall accuracy is the percent of pixels correctly simulated [46]. The Kappa coefficient serves to test whether one map differs statistically from another map (rather than simply reporting this value as another measure of accuracy) [47]. The FoM, such as the intersection over union for multiple classes, is computed by superimposing the initial land use map in 2005, the actual map in 2018, and the simulated map in 2018. The formula of the FoM is

(8) $$\mathrm{FoM}=\frac{H}{M+H+WH+FA}\times 100\%,$$

where hits H is the area where the observed and simulated land use are completely consistent, misses M is the error in the observed change simulated for persistence, wrong hits (WH) is when the predicted land use does not correspond to the observed land use, false alarms (FA) is the error of the observed persistence predicted for the change [48].

### 3.3. Scenario Setting and Forecasting

Land demands are calculated for the three scenarios, business as usual (BAU), industry priority development (IP), and urban transformation development (UT), by using the revised Markov transition probability matrix. Furthermore, the scenario development probabilities of industrial land and urban construction land are estimated based on IP and UT scenario settings. Table 1 describes the three scenarios.

To expand and invest in new factories, industrials generally choose areas with lower land prices and better traffic and terrain conditions, all hopefully near the city center. The IP scenario further highlights the impact of rail transit, location, industrial agglomeration, topography, and planning based on calibration. Specifically, the industry priority probability $P_{ip}$ is multiplied by the calibrated development probability $P_{i}$, and the generated new development probability $P_{i}^{\prime }$ acts on the selection of new industrial patch seeds and the expansion of the original industrial land. The formula is as follows:

(9) $$P_{ip}=\left(\theta_{1}\cdot D_{t}+\theta_{2}\cdot D_{c}+\theta_{3}\cdot A_{i}\right)\times \left[1-Env\right]\times \left(\theta_{4}\cdot \bar{Z_{i}}+\theta_{5}\cdot Z_{i}\right),$$

where $D_{t}$ is the distance to traffic elements such as trains, expressways, train stations, and expressway entrances and exits, $D_{c}$ is the distance to the urban center, $A_{i}$ is the aggregation level of industrial land within a specific spatial range, $\theta_{1}$, $\theta_{2}\;$, and $\theta_{3}$ are the corresponding parameters that sum to one, Env is a terrain element represented by slope, the Boolean variables $\bar{Z_{i}}$ and $Z_{i}$ are the areas without industrial planning and with the industrial planning, respectively, $\theta_{4}$ and $\theta_{5}$ are the corresponding parameters that sum to one and reflect the intensity with which the planning policy is implemented.

Under the high-intensity government policy of introducing talents, the net inflow of population hoping to obtain urban household registration will continue in urban areas. The new city residents will pursue various high-quality resources provided by the city and according to their own ability, resulting in the expansion of urban construction land that exceeds the historical trend. Due to the relatively large planned space allotted to urban construction land, no planning constraints are added to the generation of random seeds. Similarly, we multiply the urban transformation probability $P_{ut}$ by the calibrated development probability $P_{u}$ of urban built-up land to determine the way in which nature, culture, and policy combine to affect urban expansion. The newly generated development probability $P_{u}^{\prime }$ acts on the seed selection and expansion process of urban construction land. The constructed urban transformation development probability $P_{ut}$ is as follows:

(10) $$P_{ut}=\sum \upsilon_{j}R_{j}\times E_{l}^{\sigma },$$

where $R_{j}$ is a variable that characterizes the social and economic development status and public welfare level, including factors such as per capita disposable income, public budget, high-quality educational and medical resources, employment potential, and housing prices. $\upsilon_{j}$ are the weights of these factors, which sum to one, $E_{l}$ is the land resource endowment of a region, and σ is a constraint factor ranging from zero to one.

### 3.4. Assessment of Ecosystem Service and Ecological Sensitivity

For this research, we selected indicators such as water conservation service, soil conservation service, biodiversity maintenance service, soil erosion sensitivity, riverside sensitivity, and soil fertility to describe the gradient characteristics of the ecological environment. According to the Technical Guidelines for the Delineation of Ecological Protection Red Lines issued by the Ministry of Environmental Protection of China in 2017, the quantitative method of net primary productivity is used to evaluate the importance of ecological services. The general soil erosion equation and the minimal cumulative resistance model serve to evaluate the ecological sensitivity index [49]. The relevant formulas appear in Table 2.

### 3.5. Ecological Risk Index of Ecological Space Damage

To characterize the potential threat to the ecosystem of urban land expansion, we first define the regional ecological risk components and their relationships. Specifically, risk sources and ecological receptors are defined as urban land use spatial reconstruction and regional ecosystems, respectively. The stress and quantified stress factors relate to the expansion of different types of construction land and the occupation of important ecological space, respectively. The evaluation endpoint is the ecosystem services provided by the ecosystem, and the indicators of the evaluation endpoint are the loss of ecological service importance and the increase in ecological sensitivity. The occupancy of ecological space in the risk zone and the quantification of the evaluation endpoint indicators characterize the exposure level of ecological receptors and the negative impact of stress on the evaluation endpoint, respectively. The probability of occurrence of risk is represented by the ratio of urban construction land, industrial and mining land, and rural settlements to the total area of the risk zone. The ecological risk index is calculated as follows:

(11) $$ERI=\sum_{i=1}^{n}\frac{A_{r,i}}{A_{r}}\times w_{i}\left(a\bar{WR}+b\bar{S_{pro}}+c\bar{S_{bio}}+d\bar{SS}+e\bar{RS}+f\bar{SF}\right)$$

where ERI is the ecological risk index, $A_{r}$ is the total area of risk zone r, $A_{r,i}$ is the space occupied by stress factor i, and $w_{i}$ is the stress level of stress factor i. This work uses the concept of relative risk and sets the stress levels of industrial and mining land, urban construction land, and rural settlements as 3, 2, and 1, respectively. At the same time, a buffer zone of these three types of construction land is established at 1.5, 1, and 2 km, respectively, and the stress level within the buffer zone is reduced by half. $\bar{WR}$, $\bar{S_{pro}}$, $\bar{S_{bio}}$, $\bar{SS}$, $\bar{RS}$, and $\bar{SF}$ are the changes in the different ecological indicators in the risk zone, respectively, and a, b, c, d, e, and f are the corresponding weights that sum to unity.

## 4. Results

### 4.1. Revised Transition Probability Based on Markov Chain Model

The transition probability matrix and transition area matrix can be obtained by inputting the 2005 and 2018 land use classification maps into the Markov chain model. The resulting transition probability matrix reflects the likelihood of each land use type to transition to another type. The Markov transition probability matrix thus obtained for 2005–2018 is used to predict the land demand for the BAU scenario for the period of 2018–2031. At the same time, the Markov transition probability matrix is revised according to the settings for the IP and UT scenarios (see Table 3). The results show that the probability of farmland remaining unchanged from 2005 to 2018 is 0.8939, and the probability of conversion to urban construction land, rural residential land, or industrial and mining land is 0.0225, 0.0164, and 0.0431, respectively. The probabilities of converting forest land into urban construction land, rural settlements, or industrial and mining land are 0.0015, 0.0006, and 0.0048, respectively. The probability of converting rural settlements to urban construction land and industrial and mining land is 0.0539 and 0.0115, respectively. The probability of converting industrial and mining land into urban construction land and rural settlements is 0.0156 and 0.0235, respectively. Under the IP scenario, the probabilities of agricultural land, forest land, and rural settlements being converted to industrial and mining land are revised to 0.0739, 0.0082, and 0.0197, respectively. Under the UT scenario, the probabilities of agricultural land, forest land, and industrial and mining land being converted into urban construction land are 0.0486, 0.0032, and 0.0337, respectively.

### 4.2. LUCC Simulation and Sensitivity Analysis of Parameters

**Figure 2:**
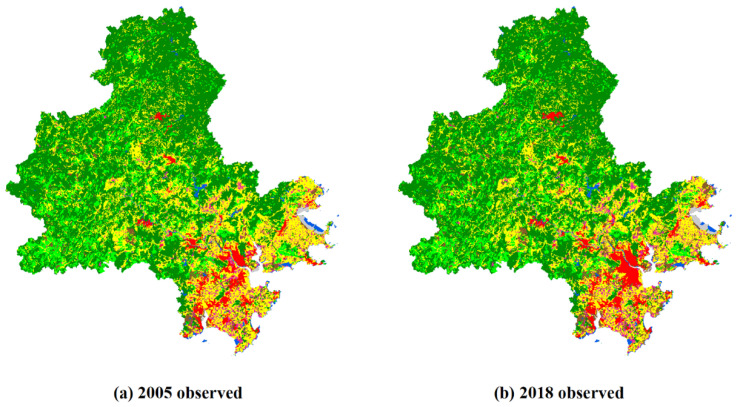

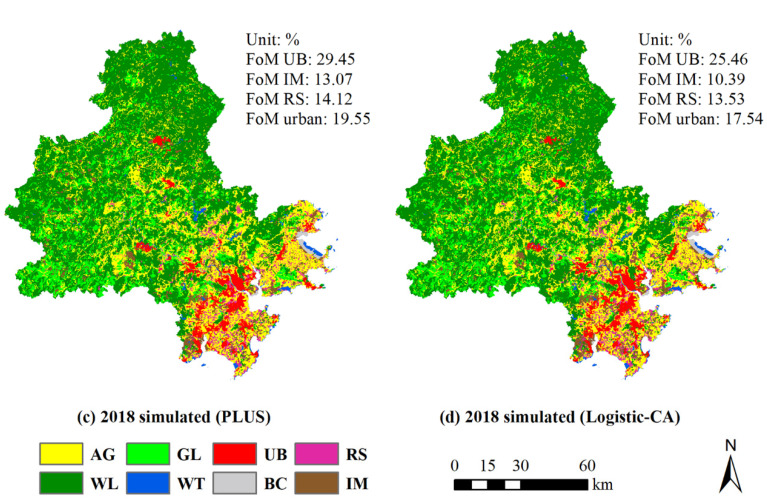
Spatial distribution of land use as simulated by PLUS model and Logistic-CA model.

To test the simulation of the PLUS model, the land use map and driving forces of Quanzhou in 2005 are input into the LEAS module to determine the probability of developing different land use types. In the random forest algorithm of the LEAS, the sampling rate is set to 0.05, the number of regression trees is 20, mTry = 9, and the number of threads is four. Furthermore, the CARS parameters such as neighborhood weight, moving window size, cost matrix, and land demand are set to simulate the spatiotemporal evolution dynamics of land use in 2018 when the percentage of seeds was 0.0001 (Figure 2). The simulation results are compared with the observational data for the years 2005 and 2018, and we calculate the accuracy indicators for the simulation such as overall accuracy, Kappa coefficient, and FoM. The results show that at a sampling rate of 0.05, the overall accuracy and the Kappa coefficient reach 91.77% and 0.878, respectively, which suggests that the simulation results of the PLUS model for the spatial distribution of land use in Quanzhou in 2018 are extremely accurate, so this model can be used to predict future land use.

To test the simulation accuracy of the PLUS model integrating the spontaneous growth mechanism of urban land expansion, we use four figures of merit, one each for urban construction land, industrial and mining land, rural settlements, and urban land, which are denoted FoM UB, FoM IM, FoM RS, and FoM urban, respectively. We then use these FoMs to compare the simulation results of the PLUS model with those of the Logistic-CA model (Figure 2). The results show that, for the PLUS model, FoM UB, FoM IM, FoM RS, and FoM urban are 29.45%, 13.07%, 14.12%, and 19.55%, respectively, which are 3.99%, 2.68%, 0.59%, and 2.01% greater than the corresponding FoMs of the organically grown Logistic-CA model. The PLUS model thus more accurately simulates the land use dynamics than the traditional urban model, which means that the PLUS model is more suitable for predicting the expansion dynamics of various types of construction land and for exploring the ecological risk faced by urban areas.

In view of the constant total target demand for a specific land type, the number of new patch seeds determined by the PoS parameter strongly affects both organic growth and spontaneous growth. To explore the way in which the number of random patch seeds in CARS affects the simulation, we conduct a sensitivity analysis of this parameter (Table 4). After step-by-step debugging, the results show that when the 0 < PoS < 0.1, the FoM for the PLUS model varies over a relatively large range. Therefore, we assign more candidate parameters in the interval 0–0.1 and fewer in the interval 0.1–1. The results show that as PoS increases from 0.0001 to 0.02, FoM UB and FoM RS decrease from 29.45% and 14.12% to 28.19% and 12.12%, respectively. Moreover, FoM IM increases from 13.07% to 16.93%, and FoM IM and FoM urban increase from 13.07% and 19.55% to 16.93% and 21.58% respectively. When PoS increases from 0.02 to 1, most FoMs decrease to a relatively narrow range and stabilize. The sensitivity of the simulation accuracy to the parameter values for different types of construction land can provide a basis for setting parameter values for different urban development scenarios.

### 4.3. Scenario Prediction of Future LUCC

Using the 2018 land use map as a starting point, the baseline and revised Markov transition probabilities are used to predict the 2031 land demand under different scenarios (Table 5). The revised coefficients $\mu_{j}$ of the Markov transition probability matrix under the industrial priority and urban transition scenarios are 1.54 and 2.16, respectively. The increase in the three types of construction land in the baseline scenario is mainly caused by the encroachment of cultivated land, whereas forest land, grassland, water bodies, and beach areas did not change significantly. Under the IP scenario, farmland, forest land, and grassland decrease, whereas the urban construction land and rural settlements do not change significantly. This indicates that the increase in industrial and mining land is mainly at the expense of farmland, forest land, and grassland. Under the UT scenario, the increase in urban construction land is at the expense of cultivated land, forest land, and industrial and mining land.

**Figure 3:**
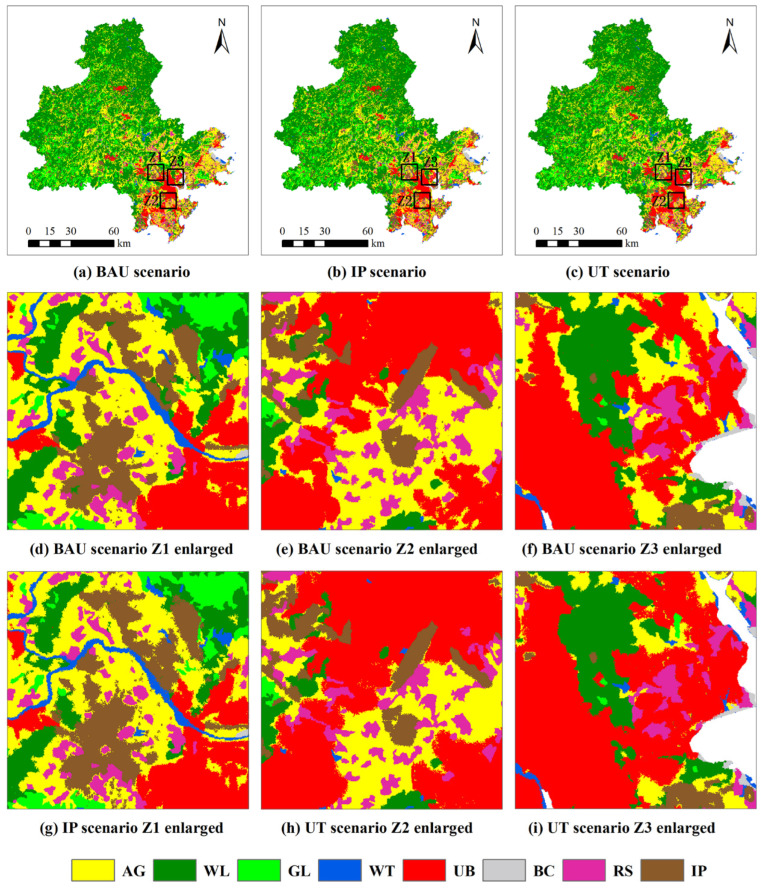
LUCC predictions for 2031 based on optimal PoS parameters (Z1: northwest of Quan-zhou Bay; Z2: east of Quan-zhou Bay; Z3: south of Quan-zhou Bay).

When simulating the spatial allocation of land use, the industrial priority probability and the urban transition probability are both multiplied by the LEAS-calibrated development probability and then used as the development potential input to CARS. According to the sensitivity to PoS parameters of construction land types (Table 4), the PoS value that provides relatively high simulation accuracy is selected as the parameter configuration for the corresponding development scenario. That is, the PoS values of CARS in the BAU, IP, and UT scenarios are set to 0.01, 0.02, and 0.0001, respectively. Figure 3 shows the land use dynamics for 2018–2031 under three development scenarios and shows three enlarged areas Z1–Z3 to the northwest, east, and south of Quanzhou Bay. The results show that the expansion of urban construction land under the UT scenario mainly occurs around Quanzhou Bay, especially in Fengze and Luojiang in the east and in Jinjiang and Shishi in the south. This is attributed mainly to the level of economic development, high-quality educational and medical resources, and good public social welfare in these regions. Under the IP scenario, the region generally experiences expansion of industrial and mining land, which is more concentrated in the periphery of urban areas. The dense distribution of expressways in the region is an important factor driving the expansion of industrial and mining land.

Table 6 predicts the increase in urban construction land and industrial and mining land under different development goals from 2018 to 2031. The results show that under the IP scenario, the industrial and mining land increase the most in Nan’an, followed by Anxi, Hui’an, and Jinjiang. Compared with the BAU scenario, the growth of industrial and mining land under the IP scenario does not differ significantly between districts and counties. The UT scenario produces significant growth in urban construction land in Jinjiang, Nan’an, and Fengze, and, relative to the BAU scenario, the rate of change of urban construction land differs strongly between districts and counties.

### 4.4. Indicators of Evaluation Endpoints

**Figure 4:**
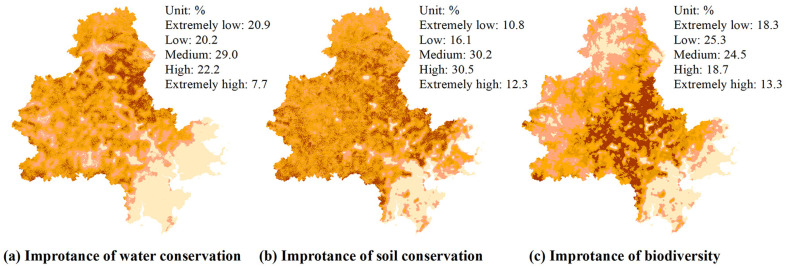

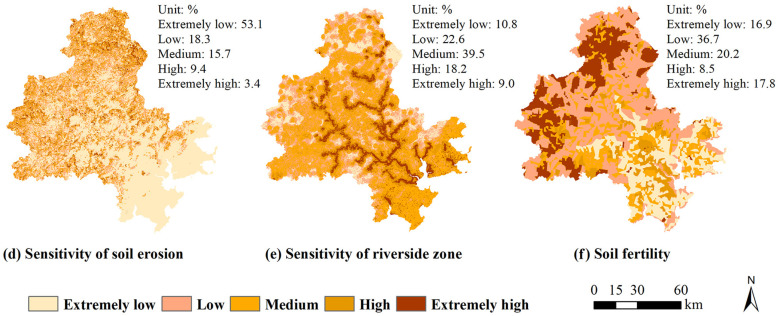
Spatiotemporal distribution of evaluation endpoints.

Using the raster calculator and cost–distance analysis tool of ArcGIS 10.6, the spatial distribution of indexes such as ecological service, ecological sensitivity, and soil fertility can be obtained by combining operation and least-cost path analysis. Furthermore, all ecological indicators are graded by applying the natural breakpoint classification method, and the indicator values are divided into five grades: extremely low, low, medium, high, and extremely high (Figure 4). Overall, the importance of ecosystem services is mainly distributed in the areas south of Daiyun Mountain and west of Qingyuan Mountain. The extreme importance of the water conservation service and soil conservation service occurs mainly in the northern region, with Dehua County and Yongchun County as the geographic centers (they occupy 7.7% and 12.3% of the study area, respectively). The extremely important areas of biodiversity appear mainly in the middle of the study area and account for 13.3% of the area. The ecological environment sensitivity is mainly distributed in the northwest of the study area and near water bodies such as rivers, lakes, and reservoirs (see Jinjiang and Luoyang Rivers). The extremely important areas of soil erosion sensitivity and riverside sensitivity account for 3.4% and 9.0% of the total area, respectively. The areas with extremely high soil fertility are mainly located in the northwest of the study area and account for 17.8% of the area.

### 4.5. Assessing Future Ecological Risk

**Figure 5:**
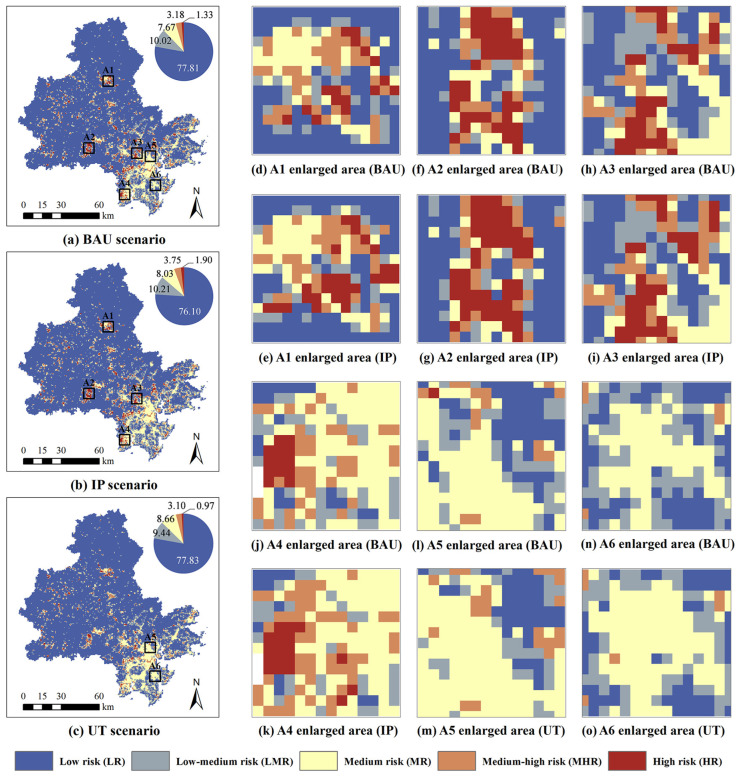
Spatial distribution in 2031 of ecological risks under different development scenarios.

Using the fishing net tool of ArcGIS, the study area was divided into a 600 m × 600 m grid, which we use as the basic spatial unit for expressing ecological risk. We calculate the ecological risk index (ERI) of each risk zone based on the land use scenarios and key ecological factors. The risk at the center of the risk unit is thus obtained and converted into raster data by using ArcGIS (Figure 5). In general, compared with the BAU scenario, the IP scenario leads to the continuous expansion of the high-risk area, the medium-high-risk area, and the medium-risk area, which account for 1.90%, 3.75%, and 8.03% of the study area, respectively. Similarly relative to the BAU scenario, the high-risk area and the medium-high-risk area of the UT scenario continue to decline. Particularly remarkable is that the medium-risk of the UT scenario expands significantly, with its area rising from 7.67% of the baseline to 8.66%. The expansion of industrial and mining lands in the central and northwestern regions produces an increase in high-risk areas (A1–A3), while the expansion of industrial and mining land in the southeast increases the medium-high-risk areas (A4). The expansion of urban construction land in the Quanzhou Bay and South Wing areas undergoes a rapid increase in medium-risk areas (A5 and A6). The industrial expansion in the southern and eastern parts of the study area (the A4 area) contributes to the development of numerous medium-high-risk areas and high-risk areas. In contrast, industrial development in the northwest region results in a significant expansion of high-risk areas. These results provide an important reference for the spatial configuration of industrial projects. The change in the spatial distribution of ecological risk in the UT scenario is mainly attributed to the fact that urban expansion mainly occurs in the Quanzhou Bay area, where the importance of ecosystem services is relatively low. At the same time, with this scenario, some industrial and mining land is converted into urban construction land.

Table 7 shows the areas with ecological risk greater than medium under different development scenarios and at the district and county scale. Comparison with Table 6 shows that under the IP scenario, the large expansion of industrial and mining land in Anxi and Nan’an in the northwest significantly increases the high-risk area. The expansion of industrial and mining land in Dehua, also located in the northwest, is relatively slow but causes a greater increase in high ecological risk. In southeastern districts and counties such as Jinjiang and Shishi, the rapid expansion of industrial and mining land has less impact on high-level ecological risks, but more on medium-level and medium-high-level risks. In the UT scenario, except for Dehua, the high-risk areas shrink in all districts and counties. In particular, the high-risk areas of Nan’an decrease significantly. Except for Fengze, Quangang, Anxi, and Yongchun, the medium- and high-risk areas of other districts and counties also decrease. The medium-risk areas increase significantly in all districts and counties under this scenario, especially Jinjiang, Nan’an, and Fengze.

## 5. Discussion

The simulation accuracy of the PLUS model is very sensitive to its parametrization (i.e., the number of random patch seeds), which means that the ratio of spontaneous growth of different types of construction land affect the accuracy of the urban model. When PoS changes from 0.0001 to 0.02, the accuracy with which rural settlements are simulated decreases by 2.0%. This is mainly attributable to the relatively small number of discontinuous expansions in rural settlements during the simulation period. At the same time, the accuracy with which urban construction land is simulated drops by 1.26%, which indicates that some discontinuous development exists with urban construction land. However, since the total predicted urban construction land is constant, the organic growth of urban construction land is somewhat disturbed. In this case, correct predictions of spontaneous urban growth do not suffice to offset the decrease in accuracy of the predicted organic growth process. This trade-off in the simulation manifests itself as a slight loss of accuracy in simulating urban built-up land. The simulation accuracy of industrial and mining land increases by 3.9%, and the simulated spontaneous growth aids the overall accuracy of the simulation. This result testifies to a broad-based discontinuous development of industrial and mining land. When PoS changes from 0.02 to 0.9999, the accuracy with which industrial land is simulated drops from its maximal value to around 16.00%, which shows that by continuously increasing the number of random patch seeds, the contribution of spontaneous growth to the simulation accuracy has difficulty offsetting the error introduced by organic growth. Another possible explanation is that the predicted scale of the spontaneous growth of industrial land exceeds the historical observations of spontaneous growth [26], which would also cause the spontaneous growth to degrade the simulation. For similar reasons, the accuracy with which urban construction land and rural settlements are simulated decreases when PoS changes from 0.02 to 0.9999.

Investigating possible future development trajectories of cities will provide credible evidence for the spatial distribution of risk sources. The simulation results indicate that significant differences in urban form appear under different development scenarios, and these differences are mainly reflected in the two categories of urban construction land and industrial and mining land. The past development of different types of construction land and the factors that drive such development are fundamentally responsible for the differences in urban form. In the UT scenario, Jinjiang and Shishi in Quanzhou Bay and southern regions gain rapid development. These regions offer more advantages in economic development, social welfare, and educational and medical resources compared with other districts and counties. Since the historical development of urban construction land remains extremely compact, the setting of spontaneous growth and urban transition probability guides urban expansion to the southeast. In other words, the UT scenario provides more development opportunities to the southeastern non-urban areas with superior potential, and as a result, the density of urban construction land decreases somewhat. In the IP scenario, industrial and mining land expands widely in areas with developed traffic and close to urban centers. Due to the high-speed network reaching every district and county, highway entrances and exits are spread throughout the region, which is one of the most important forces driving industrial development. Therefore, the expansion pattern of industrial and mining land reflects the characteristics of local agglomeration but remains relatively discrete over the whole area. These differences in the development of urban spatial forms cause the increased development of construction land in the UT and IP scenarios to differ completely on the district and county scale.

Compared with the BAU scenario, the ecological risks predicted by both the UT and IP scenarios changed significantly. Although the industrial and mining land in the IP scenario increases by 20% with respect to the BAU scenario, the high-risk area increases by 42.5%. Under the UT scenario, the urban construction land increases by 20% compared with the BAU scenario, and the change in risk is mainly reflected in the 13% expansion of the medium-risk area. In the future, both medium and high ecological risk in Quanzhou Bay and the northwest will continue to expand.

The expansion of different types of construction land may produce different ecological risks in different local spaces, so it is vital to explore ways to protect ecological resources and reduce ecological risk. On the one hand, reducing the development density and controlling the development scale of urban construction land in Quanzhou Bay should reduce such risk. On the other hand, the newly added industrial land incurs a relatively low risk in the southeast, and it is extremely urgent to guide the development of industrial land in this area. Given that all districts and counties possess the inherent motivation to develop industrial projects to promote rapid economic development, the planning of industrial land should adopt a more binding implementation strategy. At the same time, given that construction land is mainly developed in the southeast, the development space, timing, and scale in towns of the same size require scientific demonstration. The urban form and ecological risks predicted herein provide a spatially quantified basis for trade-offs in formulating and implementing planning strategy.

In a continuous space in a large area, the availability of evaluation data superimposes the compound influence of natural and human factors, making regional ecological risk assessment an important challenge. By adopting a greater spatial resolution, more classification of construction land, and more accurate LUCC prediction models, more accurate spatial information on stress sources can be obtained. At the same time, the spatiotemporal gradient characteristics of regional ecosystems are described from the perspectives of ecosystem services and ecological sensitivity, so the evaluation endpoint indicators can be accurately quantified. Based on these scientific foundations, we explore the way in which construction land expansion affects ecological risks and their spatial differentiation in rapidly urbanizing regions. Such risk assessment is based on the following assumptions: A greater development scale or frequency of the “urbanization source” of construction land corresponds to a greater possibility of stressing ecological receptors. Next, the characteristics of the evaluation endpoint of ecological receptors, including the service type and provision capacity of ecosystem services and the level of ecological sensitivity, are closely related to the availability of the ecological space on which they depend. Finally, the severity of the negative effects of stress on evaluation endpoints depends on relative exposure and the endpoint characteristics.

We now discuss the more complex factors that affect the ecological risk that may arise from development activities. Does the intensity of changes in ecological risk differ from location to location? These issues still need to be addressed in future research. From individual to planetary scales, nonlinear stress response relationships such as adaptive, dynamic, and interactive commonly occur [50]. Such ecological nonlinearity clearly explains the occurrence and development of ecological risk. For example, in high-density industrialized or urbanized areas with strict environmental regulations, strong citizen awareness of environmental protection, and excellent social management capabilities, specific construction land, and human activities on it do not necessarily cause ecological risks. In some less-developed regions, the situation is often the exact opposite. At the same time, Darwinian ecological fitness explains that the long-term presence of low-dose stress in a specific spatiotemporal context may not always negatively affect receptors [51]. Considering the inherent nonlinearity of the stress influence relationship in ecological risk assessment, more in-depth research into regional ecological risk is urgent.

In the context of the path dependence of the development of the spatial form of urban land use, the Markov method is a reliable method for predicting land demand in the relatively near future (several decades) [38,52]. It makes the important assumption that the future transformation of urban land use is based on historical land use and is significantly affected by the transformation of historical land use. However, the question arises of whether the speed of urban expansion remains constant from the historical time frame to the future time frame. In particular, when the time span is significantly enlarged, the speed of urban expansion may change [28]. This allows the Markov method used herein to improve the accuracy of predictions of BAU scenarios. 

The speed of urban expansion is driven or constrained by various factors such as population, the economy, and natural ecology [53,54]. In [39], a multiple regression model between urban area, population, and GDP was used to improve the prediction of land demand and land use conversion by applying Markov chains. In contrast, the present work examines a more detailed classification of construction land. Therefore, the following issues should be considered a priority when predicting the future development of construction land: (1) How does the integration of social, economic, and ecological models help predict the future population, GDP, and environmental carrying capacity of urban areas? (2) Given these socio-economic and ecological factors, how do we predict the speed and scale of expansion of different types of construction land? (3) Finally, how do we correct the Markov transition probability matrix during the forecast period to obtain the future land conversion demand of different types of land?

## 6. Conclusions

This research reveals the way in which the spatial reconstruction of different types of construction land affects the temporal and spatial evolution of regional ecological risk. First, taking Quanzhou City as the study area and using the Markov chain method, the land use transition probability matrix under different urban development orientations was revised to predict land demand. Next, the contribution of land use expansion is intelligently mined based on the random forest algorithm, and the organic growth and spontaneous growth of land use are simulated by CA based on multi-type random patch seeds. Finally, the key ecosystem services and ecological sensitivity indicators are screened to predict areas of intense ecological risk given the spatial stress produced by various types of construction land.

The results show that (1) land use change can be simulated by the PLUS model effectively, and the simulation results are sensitive to the parametrization (i.e., the number of random patch seeds). The overall accuracy and Kappa coefficient reach 91.77% and 0.878, respectively. The urban construction land for PoS = 0.0001 has the highest FoM, which is 3.9% higher than that produced by the Logistic-CA model. The accuracy with which industrial and mining land is simulated with PoS = 0.02 is 6.5% greater than that of the Logistic-CA model. (2) Since the temporal and spatial evolution paths of different types of construction land differ significantly, the future urban forms of different development scenarios will have significant spatial differences. Under the UT scenario, urban construction land is mainly concentrated in the Quanzhou Bay area, whereas the southern areas such as Jinjiang and Shishi have high development potential. Industrial and mining land continues to expand mainly in suburbs with good traffic conditions and proximity to town centers. Nan’an, Anxi, and Hui’an are the key areas for the continuous expansion of industrial and mining land in the future. (3) In the future, the spatial expansion of construction land in Quanzhou will lead to medium and high ecological risks. Compared with the BAU scenario, the industrial and mining land area increases by 20% under the IP scenario, whereas the high ecological risk increases by 42.5%. At the same time, the medium-high and medium risks in this scenario also continue to increase. Under the UT scenario, the urban development of Quanzhou Bay and the southern region mainly leads to the agglomeration and increase in medium-level ecological risk. Compared with the BAU scenario, the high- and medium-high risks continue to decline in the UT scenario. The results provide a scientific reference for implementing territory development plans and formulating risk management policies. Given the nonlinear relationships between stress and effects, the question of how to scientifically characterize regional-scale nonlinear ecological risks requires further exploration and research.

## Figures and Tables

**Table 1:** Setting of urban expansion scenarios based on CARS.

Scenario Type	Description
BAU	Based on historical trends and driving factors from 2005 to 2018, this scenario uses Markov chains to forecast land demand from 2018 to 2031. At the same time, the development probabilities of various land uses obtained during the calibration are used to control the micro-spatial allocation process of CARS.
IP	As a high-speed industrial development goal that considers government planning and rail transit orientation, the IP scenario sets a 20% increase in the target value of industrial and mining land compared to the BAU scenario. The scenario uses a revised Markov transition probability matrix to forecast land demand in 2018–2031. At the same time, the designed IP development probability is multiplied by the calibration probability to obtain the development potential parameters of industrial land in CARS.
UT	This is a high-speed urban expansion scenario oriented to attract high-end talents under a high degree of government intervention. The amount of urban construction land set in the UT scenario is 20% higher than the BAU scenario. Similarly, the revised Markov transition probability matrix is used to forecast land demand in 2018–2031. Considering the level of economic development and the spatial distribution of high-quality public resources, the designed UT development probability is multiplied by the calibration probability to obtain the development potential parameters of urban construction land in CARS.

**Table 2:** Formulas for calculating the importance of ecosystem services and the sensitivity of the ecological environment.

Indicator	Formula	Description
Water conservation service (WR)	$WR=NPP_{mean}\times F_{sic}\times F_{pre}\times \left(1-F_{slo}\right)\;\;$	Water conservation service is a process in which ecosystems redistribute precipitation to effectively regulate water flow and water cycle. $NPP_{mean}$ is the average annual net primary productivity, $F_{sic}$ is the soil seepage factor, $F_{pre}$ is the annual average precipitation, $F_{slo}$ is the slope factor.
Soil conservation service ($S_{pro}$)	$S_{pro}=NPP_{mean}\times \left(k-1\right)\times \left(1-F_{slo}\right)$	Soil conservation service is the ability of an ecosystem through its structure and processes to reduce soil erosion due to rainfall or runoff. k is the soil erodibility factor.
Biodiversity maintenance service ($S_{bio}$)	$S_{bio}=NPP_{mean}\times F_{pre}\times F_{tem}\times \left(1-F_{alt}\right)\;\;$	Biodiversity maintenance services are the ability of an ecosystem to maintain the diversity of genes, species, habitats, communities, and ecological processes. $F_{pre}$ is the annual average precipitation, $F_{tem}$ is the annual average temperature, $F_{alt}$ is the altitude factor.
Sensitivity of soil erosion (SS)	$SS_{i}=\sqrt[{4}]{R_{i}\times K_{i}\times LS_{i}\times C_{i}}$	Sensitivity of soil erosion is the possibility of soil and its parent material being destroyed, denuded, transported, and deposited under the action of natural external forces dominated by hydrodynamics. $R_{i}$ is rainfall erosivity, $K_{i}$ is soil erodibility, $LS_{i}$ is slope length and slope, and $C_{i}$ is vegetation cover on the ground.
Sensitivity of riverside zone (RS)	$MCR=f_{min}\overset{i=m}{\underset{j=n}{\sum }}\left(D_{ij}\times R_{i}\right)$	Sensitivity of riverside zone is the sensitive response and self-recovery ability of the transition zone between rivers and land under natural and man-made external disturbances. MCR is the minimum cumulative resistance value, $D_{ij}$ is the spatial distance of the evaluation target from source j to landscape unit i, $R_{i}$ is the resistance value of landscape unit i, $f_{min}$ represents the positive correlation between the minimum accumulated resistance and the variables $D_{ij}$ and $R_{i}$.
Soil fertility (SF)	SF=TN×0.15+TP×0.3+TK×0.4+TOM×0.15	Soil fertility characterizes the ecological adaptability of soil. TN is total nitrogen, TP is total phosphorus, TK is total potassium, TOM is total organic matter.

**Table 3:** The Markov transition probability matrices for three evolutionary scenarios over the period of 2018–2031.

Evolutionary Scenarios	Probability of Changing to the Following Land Use:							
	AG	WL	GL	WT	UB	BC	RS	IM
2018–2031 (BAU)								
AG	0.8939	0.0164	0.0062	0.0013	0.0225	0.0002	0.0164	0.0431
WL	0.0077	0.9754	0.0096	0.0004	0.0015	0.0000	0.0006	0.0048
GL	0.0084	0.0280	0.9516	0.0003	0.0014	0.0000	0.0015	0.0086
WT	0.0207	0.0082	0.0043	0.8930	0.0034	0.0530	0.0033	0.0142
UB	0.0124	0.0036	0.0054	0.0010	0.9726	0.0001	0.0019	0.0030
BC	0.0255	0.0011	0.0001	0.0049	0.0026	0.8991	0.0044	0.0623
RS	0.0389	0.0040	0.0018	0.0019	0.0539	0.0004	0.8875	0.0115
IM	0.0248	0.0138	0.0064	0.0063	0.0156	0.0009	0.0235	0.9087
2018–2031 (IP)								
AG	0.8652	0.0159	0.0060	0.0013	0.0218	0.0002	0.0159	0.0739
WL	0.0077	0.9720	0.0096	0.0004	0.0015	0.0000	0.0006	0.0082
GL	0.0083	0.0278	0.9457	0.0003	0.0014	0.0000	0.0015	0.0147
WT	0.0205	0.0081	0.0043	0.8838	0.0034	0.0525	0.0033	0.0243
UB	0.0124	0.0036	0.0054	0.0010	0.9705	0.0001	0.0019	0.0051
BC	0.0243	0.0010	0.0001	0.0047	0.0025	0.8565	0.0042	0.1068
RS	0.0386	0.0040	0.0018	0.0019	0.0535	0.0004	0.8801	0.0197
IM	0.0248	0.0138	0.0064	0.0063	0.0156	0.0009	0.0235	0.9087
2018–2031 (UT)								
AG	0.8701	0.0160	0.0060	0.0013	0.0486	0.0002	0.0160	0.0420
WL	0.0077	0.9737	0.0096	0.0004	0.0032	0.0000	0.0006	0.0048
GL	0.0084	0.0280	0.9501	0.0003	0.0030	0.0000	0.0015	0.0086
WT	0.0206	0.0082	0.0043	0.8895	0.0073	0.0528	0.0033	0.0141
UB	0.0124	0.0036	0.0054	0.0010	0.9726	0.0001	0.0019	0.0030
BC	0.0254	0.0011	0.0001	0.0049	0.0056	0.8964	0.0044	0.0621
RS	0.0363	0.0037	0.0017	0.0018	0.1163	0.0004	0.8289	0.0107
IM	0.0243	0.0135	0.0063	0.0062	0.0337	0.0009	0.0231	0.8920

Note: AG: Agricultural, WL: Woodland, GL: Grassland, WT: Water, UB: Urban built-up, BC: Beach, RS: Rural settlement, IM: Industrial and mining land.

**Table 4:** Sensitivity of FoMs of construction land to the number of random seeds (PoS).

PoS	FoM (%)				PoS	FoM (%)			
	UB	IM	RS	Urban		UB	IM	RS	Urban
0.0001	29.45	13.07	14.12	19.55	0.06	27.34	16.23	11.95	20.91
0.001	29.11	13.46	14.04	19.73	0.07	27.27	16.22	12.06	20.94
0.005	28.58	14.82	13.12	20.33	0.08	27.20	16.08	11.89	20.81
0.01	28.31	15.75	12.77	20.87	0.09	27.26	16.04	12.03	20.68
0.02	28.19	16.93	12.12	21.58	0.1	27.34	16.11	11.92	20.85
0.03	27.63	16.70	11.96	21.24	0.5	27.12	15.96	12.03	20.70
0.04	27.46	16.26	11.98	20.96	0.999	27.14	16.01	11.75	20.72
0.05	27.44	16.22	11.99	20.96	0.9999	27.14	15.98	11.93	20.72

**Table 5:** Land demand in 2031 in Quanzhou under different development scenarios (units: ha).

Land Use Type	2018	BAU Scenario	IP Scenario	UT Scenario
		2031	2031	2031
AG	246,167.64	229,085.55	221,993.37	223,121.52
WL	553,564.17	549,437.22	547,411.14	548,387.01
GL	158,190.3	158,029.47	157,015.17	157,703.85
WT	14,150.07	13,633.92	13,515.57	13,589.55
UB	45,880.29	53,397.09	53,092.8	64,036.44
BC	6286.14	6537.78	6232.05	6486.93
RS	34,193.07	36,587.79	36,195.12	34,428.42
IM	44,488.89	56,211.75	67,436.28	55,161.18

**Table 6:** Construction land expansion at district and county scale during 2018–2031.

County	Increment of BAU Scenario (2018–2031)		Increment of IP Scenario (2018–2031)		Increment of UT Scenario (2018–2031)	
	UB (ha)	IM (ha)	IM (ha)	Rate of Change Compared to the BAU Scenario (%)	UB (ha)	Rate of Change Compared to the BAU Scenario (%)
Licheng	344.7	18	29.34	1.63	640.44	1.86
Fengze	618.84	240.84	338.4	1.41	1720.62	2.78
Luojiang	207.54	444.06	863.01	1.94	556.02	2.68
Quangang	414.18	687.69	1402.47	2.04	1045.44	2.52
Hui’an	692.91	2045.34	3885.3	1.90	1540.26	2.22
Anxi	474.66	2112.03	4249.08	2.01	640.62	1.35
Yongchun	259.11	769.86	1549.44	2.01	497.7	1.92
Dehua	197.55	486.99	954.72	1.96	203.04	1.03
Shishi	483.66	637.2	1204.38	1.89	1166.76	2.41
Jinjiang	2251.17	1531.98	2656.53	1.73	6308.46	2.80
Nan’an	2051.01	2721.15	5787	2.13	4315.32	2.10

Note: The spatial range simulated in this research does not include Kinmen County.

**Table 7:** Ecological risks greater than medium in all districts and counties in 2031.

County	Increment of BAU Scenario (2031)			Increment of IP Scenario (2031)			Increment of UT Scenario (2031)		
	MR (ha)	MHR (ha)	HR (ha)	MR (ha)	MHR (ha)	HR (ha)	MR (ha)	MHR (ha)	HR (ha)
Licheng	3168	792	36	3168	756	36	3780	468	36
Fengze	4356	936	0	4284	1080	144	5436	1044	0
Luojiang	2232	1188	900	2484	1404	1152	2484	1152	468
Quangang	3852	1584	216	3636	2052	720	3744	1800	0
Hui’an	8316	3744	504	9216	4932	720	8820	3420	180
Anxi	10,008	6084	5112	10,584	7452	6840	10,836	6912	4536
Yongchun	3924	2340	1440	4248	3168	1692	4032	2664	1188
Dehua	2880	2196	1008	3096	2160	1656	2844	2160	1404
Shishi	4284	936	108	4464	1332	108	4896	900	0
Jinjiang	21,168	3924	252	22,032	4392	468	25,632	3636	108
Nan’an	20,484	11,376	5148	21,456	12,672	7452	23,148	10,080	2844

Note: The area simulated in this research does not include Kinmen County.

## Data Availability

Not applicable.

## Abbreviations

LUCC	Land use and land cover change
ERI	Ecological risk index
CA	Cellular automata
GIS	Geographical information system
GDP	Gross Domestic Product
PLUS	Patch-generating land use simulation model
CARS	CA model based on multi-type random patch seeds
LEAS	Land use expansion analysis strategy
PoS	Percent-of-seeds
BAU	Business as usual
IP	Industry priority development
UT	Urban transformation development
FoM	Figure of merit
WH	Wrong hits
FA	False alarms
AG	Agricultural
WL	Woodland
GL	Grassland
WT	Water
UB	Urban built-up
BC	Beach
RS	Rural settlement
IM	Industrial and mining land
LR	Low risk
LMR	Low-medium risk
MR	Medium risk
MHR	Medium-high risk
HR	High risk
